# Motility and stem cell properties induced by the epithelial-mesenchymal transition require destabilization of lipid rafts

**DOI:** 10.18632/oncotarget.9928

**Published:** 2016-06-09

**Authors:** Michael J. Tisza, Weina Zhao, Jessie S.R. Fuentes, Sara Prijic, Xiaoling Chen, Ilya Levental, Jeffrey T. Chang

**Affiliations:** ^1^ Department of Integrative Biology and Pharmacology, The University of Texas Health Science Center at Houston, Houston, TX, USA; ^2^ Department of Bioinformatics and Computational Biology, The University of Texas MD Anderson Cancer Center, Houston, TX, USA

**Keywords:** epithelial-mesenchymal transition, cell motility, stem cell properties, plasma membrane, lipid rafts

## Abstract

The Epithelial-Mesenchymal Transition (EMT) is a developmental program that provides cancer cells with the characteristics necessary for metastasis, including increased motility and stem cell properties. The cellular and molecular mechanisms underlying this process are not yet fully understood, hampering efforts to develop therapeutics. In recent years, it has become apparent that EMT is accompanied by wholesale changes in diverse signaling pathways that are initiated by proteins at the plasma membrane (PM). The PM contains thousands of lipid and protein species that are dynamically and spatially organized into lateral membrane domains, an example of which are lipid rafts. Since one of the major functions of rafts is modulation of signaling originating at the PM, we hypothesized that the signaling changes occurring during an EMT are associated with alterations in PM organization. To test this hypothesis, we used Giant Plasma Membrane Vesicles (GPMVs) to study the organization of intact plasma membranes isolated from live cells. We observed that induction of EMT significantly destabilized lipid raft domains. Further, this reduction in stability was crucial for the maintenance of the stem cell phenotype and EMT-induced remodeling of PM-orchestrated pathways. Exogenously increasing raft stability by feeding cells with ω-3 polyunsaturated fatty acid docosahexaenoic acid (DHA) repressed these phenotypes without altering EMT markers, and inhibited the metastatic capacity of breast cancer cells. Hence, modulating raft properties regulates cell phenotype, suggesting a novel approach for targeting the impact of EMT in cancer.

## INTRODUCTION

The epithelial to mesenchymal transition (EMT) is a latent developmental program that, in cell culture models, can be triggered by an array of signals, such as the ectopic expression of developmental transcription factors such as snail or twist [1, 2], or activation of growth factor pathways, including TGF-β [3, 4]. These triggers induce a large-scale reprogramming of cellular gene expression and signaling, leading to a loss of epithelial markers such as e-cadherin and gain in mesenchymal markers including vimentin and n-cadherin [5, 6]. When activated in cancer cells, this mesenchymal cellular program increases resistance to chemotherapeutics, cell motility, invasion, and the ability to seed new tumors [5, 7]. While its involvement in human tumors remains controversial, there is increasing evidence that EMT is sufficient, even if not necessary, for metastasis [8–10].

Concomitant with the acquisition of an aggressive phenotype, the EMT is marked by a profound rewiring of the cell signaling programs that affect a multitude of pathways [11, 12]. Many (including EGFR, WNT, and HER2) are activated by extracellular ligands or receptors located at the plasma membrane (PM), suggesting that changes in the properties of the PM may facilitate the wholesale signaling network rearrangements associated with an EMT. Supporting this possibility, the lipid compositions of cells in epithelial or mesenchymal states have been shown to be distinct [13] and useful in distinguishing cells with an EMT phenotype [14]. Further, we have shown that alterations in the fluidity of the plasma membrane, e.g. by modulating cholesterol content, can induce or inhibit an EMT, although the impact of these changes on the organization of the PM is still not known [15].

A potential consequence of the remodeling of the membrane lipidome observed in an EMT [14] is a lateral reorganization of cellular membranes, specifically the PM. A core feature of PM physiology is its subdivision into compositionally and functionally distinct signaling microdomains, such as lipid rafts [16]. Rafts are dynamic lipid and protein assemblies driven by the physicochemical differences between two distinct membrane states: a liquid-ordered (raft) domain rich in sphingolipids, saturated lipids, and cholesterol, and a disordered (non-raft) domain enriched in unsaturated lipids [17]. This distinction in membrane states can be observed in plasma membranes isolated from cultured cells, termed Giant Plasma Membrane Vesicles (GPMV), a model that maintains the compositional complexity of cell PMs (compared to models employing artificial membranes) and separates into microscopic raft and non-raft domains [18, 19]. Most importantly, this system allows quantitative investigation of raft-related phenomena, such as the composition and stability of coexisting membrane domains [20–22] and the factors governing their physical properties [21, 23, 24].

Because many signaling programs rewired by EMT are routed through the PM, we hypothesized that alterations in PM biophysical properties accompany EMT. Additionally, since membrane properties are central to cell function, we hypothesize that reversing the EMT-induced changes to membrane structure would also repress malignant EMT phenotypes. Modulation of raft domains is a therapeutically attractive approach because a number of pharmacological and nutritional factors have been shown to influence raft stability and function [25–27]. Notable among these is the modulation of membrane structure by ω-3 polyunsaturated fatty acid docosahexaenoic acid (DHA), which we have recently discovered to stabilize raft domains in a range of cell culture models [28]. (Levental KR, Surma MA, Lorent JH, Klose C, Chen C, Chang JT, Levental I. ω −3 polyunsaturated fatty acids direct differentiation of the membrane phenotype in mesenchymal stem cells to potentiate osteogenesis. Unpublished). DHA is an essential fatty acid that is broadly beneficial in a number of pathophysiological conditions, including cardiovascular disease [29] and cancer [30]. The molecular mechanisms underlying these effects are currently unclear, although one hypothesis is that DHA incorporates into membrane lipids and affects the properties of PM microdomains [31, 32].

Here, we investigated the impact of PM microdomains on EMT and observed that irrespective of the mode of induction, EMT destabilized raft domains. When domain destabilization was reversed by feeding with physiologically relevant levels of DHA [33, 34], cells undergoing EMT reverted to a more epithelial phenotype with respect to motility and stem cell properties, although they continued to exhibit mesenchymal markers. Stabilization of raft domains also shifted the signaling programs of cell surface receptors back towards an epithelial state, consistent with a model where PM properties can direct these programs. Taken together, these results demonstrate that the PM phenotype is a key determinant of the transcriptional and phenotypic outputs of EMT, and thus may be a target for therapeutic strategies targeting the metastatic potential driven by EMT in cancer.

## RESULTS

### EMT reduces raft phase stability in GPMVs

The EMT is associated with large-scale transcriptional reprogramming and changes in plasma membrane composition [12–14]. Because EMT affects a wide range of cell signaling pathways originating from the PM, we hypothesized that these effects may be associated with altered plasma membrane microdomain organization. To investigate this, we selected the human mammary epithelial cell line (HMLE) that is commonly used in studies of EMT [35]. We induced EMT in these cells by stimulation with TGF-β or ectopic expression of the transcription factors snail (SNAI1), twist (TWIST1), or slug (SNAI2). After seven days of induction, the cell morphology changed from the cobblestone appearance characteristic of epithelial cells to the spindle-like morphology indicative of mesenchymal cells (Supplementary Figure 1A). To confirm an EMT, we quantified the expression of key genes and observed that the epithelial markers claudin 1 (*CLDN1*) and e-cadherin (*CDH1*) were downregulated, while the mesenchymal markers vimentin (*VIM*) and n-cadherin (*CDH2*) were induced (Supplementary Figure 1B).

**Figure 1:**
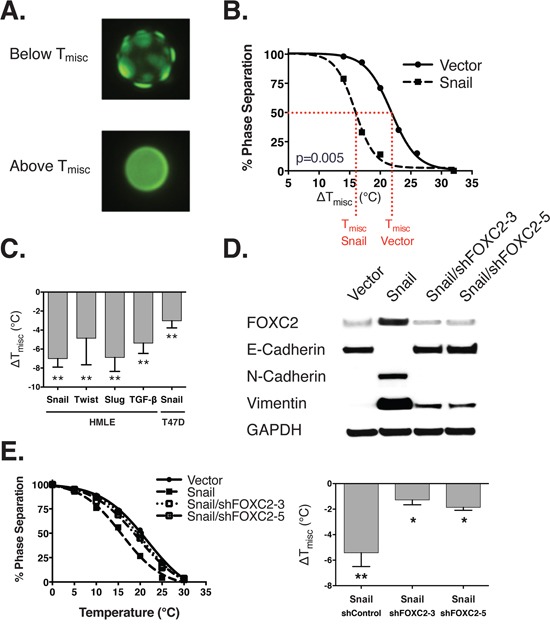
The epithelial-mesenchymal transition leads to destabilized phase separation **A.** GPMVs below the miscibility transition temperature (T_misc_) (top) exhibit distinct separation into coexisting raft and non-raft phases. The GPMVs above T_misc_ are more microscopically uniform. **B.** The relationship between temperature (x-axis) and phase separation (y-axis) is shown as sigmoidal fitted curves. T_misc_ is the temperature at which 50% of the GPMVs are phase separated (shown by the dotted red line). Δ T_misc_ is the difference in T_misc_ between HMLE cells ectopically expressing either a vector control or snail. **C.** This shows the Δ T_misc_ of HMLE and T47D cells (relative to a vector or vehicle control) induced to undergo EMT using a range of different EMT inducers (x-axis). **D.** This immunoblot shows the expression of FOXC2, epithelial (e-cadherin), and mesenchymal (n-cadherin, vimentin) markers in HMLE cells with and without ectopic expression of Snail, and shRNA knockdown of FOXC2. From left to right: HMLE/Vector, HMLE/Snail/shControl, HMLE/Snail/shFOXC2-3, and HMLE/Snail/shFOXC2-5. **E.** Under the same conditions, we counted the percent of cells exhibiting phase separation (left panel) and calculated the change in Tmisc under each condition (right panel). *p*-values are indicated: * *p*<0.05, ** *p*<0.01, *** *p*<0.001, **** *p*<0.0001, NS *p*≥0.05.

To assess changes in plasma membrane organization during an EMT, we isolated PMs from HMLE cells as GPMVs and quantified raft phase stability. We varied the temperature and visualized the separation of raft and non-raft domains (Figure 1A, Supplementary Figure 2). At lower temperatures, liquid-liquid domain separation is microscopically observable by the preferential partitioning of a non-raft phase dye [23]; at higher temperatures, the GPMVs are microscopically uniform. The temperature at which the domains observed in the GPMVs shift from a separated to uniform state is a biophysical property that is related to the composition and structure of the rafts, and can reveal differences in the PM organization across cell states.

We quantify *raft domain stability* by measuring the temperature at which 50% of the vesicles are phase separated, i.e. the miscibility transition temperature (T_misc_) [21, 24]. This temperature is proposed to be related to the size and lifetime of ordered domains at physiological temperatures [36], and thus can be interpreted as a measurable parameter related to the stability of raft domains. GPMVs isolated from HMLE cells induced to undergo EMT showed phase miscibility at significantly lower temperatures compared to controls (Figure 1B-1C, Supplementary Figure 3), indicating that cells in the epithelial state possess more stable lipid raft domains. Qualitatively similar effects were observed for EMT induced by three separate transcription factors and TGF-β stimulation, and was confirmed in the T47D breast cancer cell line, cultured in medium with serum, in contrast to the defined medium used for HMLE cells (Figure 1C).

To test whether a mesenchymal-to-epithelial transition (MET) would induce the opposite effect on the stability of lipid raft domains, we induced an MET by shRNA knockdown of FOXC2, which we previously discovered to be a master regulator of the EMT, in the HMLE/Snail cells [37]. This resulted in re-expression of epithelial markers accompanied by raft stabilization (Figure 1D-1E). Taken together, these observations demonstrate that the EMT is correlated with the stability of raft domains across EMT inducers, cell line models, and growth conditions.

### Alterations in EMT markers precede raft phase changes

**Figure 2:**
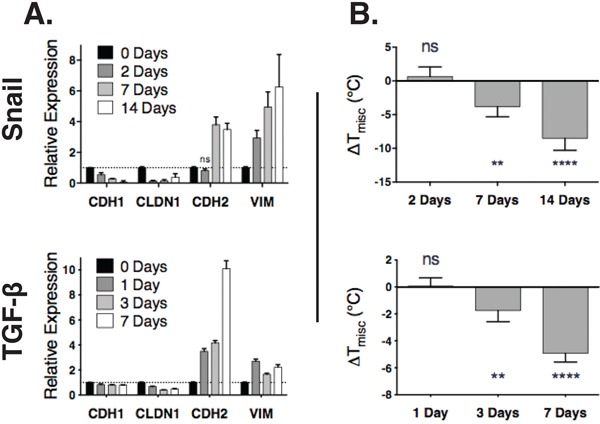
Changes in an EMT transcription program precede lipid raft destabilization **A.** Gene expression (y-axis) of epithelial (*CDH1, CLDN1*) and mesenchymal (*CDH2, VIM*) markers are measured by RT-qPCR after induction of EMT in an ER-inducible Snail model (top) or by TGF-β (bottom). Except for one (labeled), all other expression values, differ from the 0-day time point with *p* < 0.05. **B.** Raft stability (y-axis) normalized to the 0 day time point over time (x-axis) after introduction of an EMT inducer (Snail, top; TGF-β, bottom).

To investigate whether the raft changes were a driver or consequence of an EMT, we performed a time course experiment. HMLE cells were induced to undergo an EMT using an ER-inducible snail model [5]. GPMVs and RNA were collected 0, 2, 7, and 14 days after induction of snail. Within two days, transcriptional changes characteristic of EMT were detected, and by seven days, mesenchymal markers were significantly upregulated (Figure 2A). However, changes to phase separation stability were detectable only at the seventh day of treatment (Figure 2B). We repeated this experiment using TGF-β to induce EMT, and saw again a significant change in both EMT markers and raft stability by day 7. In both cases, the EMT markers changed before any detectable changes in raft stability. The fact that the changes in raft stability occurred consequent to alterations in EMT markers suggests that this property is not a prerequisite for the induction of an EMT.

### Stabilization of raft phase separation inhibits EMT properties

**Figure 3:**
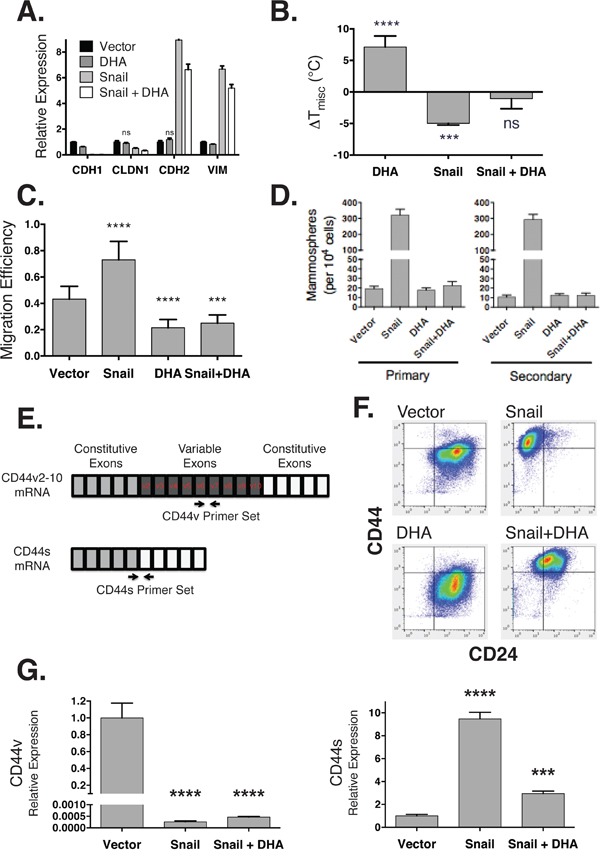
DHA maintains a mesenchymal state while inhibiting stem cell phenotypes **A.** Gene expression (y-axis) for epithelial (*CDH1*, *CLDN1*) and mesenchymal (*CDH2*, *VIM*) markers is measured by RT-qPCR in HMLE cells expressing a control vector (*Vector*), treated with 20 μM DHA (*DHA*), expressing Snail (*Snail*), or both expressing Snail and treated with DHA (*Snail + DHA*). Except for two markers (labeled), all other expression values, differ from the 0-day time point with *p* < 0.05. **B.** Raft stability (y-axis) normalized to vector control. **C.** Cell migration (y-axis) measured using a scratch-wound assay. **D.** Number of primary (left) and secondary (right) mammospheres formed in each condition. **E.** CD44 is alternatively spliced into a long variant (CD44v) isoform in epithelial cells, and a standard (CD44s) isoform in mesenchymal cells. **F.** Scatter plots of expression of cell surface markers CD24 (x-axis) and CD44 (y-axis) in HMLE cells expressing snail (right panels) or treated with DHA (bottom panels). **G.** The relative expression of CD44v (left) and CD44s (right) isoforms in HMLE cells with Snail and 20 μM DHA treatment.

Because raft stability was significantly reduced following an EMT, we hypothesized that these changes were important to maintain an EMT phenotype. If correct, this hypothesis would predict that stabilizing raft domains would inhibit or reverse acquired EMT characteristics. To test this hypothesis, we stabilized raft domains by supplementing cells with DHA. This essential ω-3 polyunsaturated fatty acid (PUFA) metabolically incorporates into membrane lipids and induces their recruitment into non-raft domains phase due to the bulky nature of the polyunsaturated acyl chain [38]. It is hypothesized that enrichment of these bulky lipids in the non-raft domain increases its disorder, ultimately stabilizing rafts by inhibiting mixing of the two phases. After treating cells that have undergone an EMT with 20 uM DHA, we observed only minor changes in EMT markers (Figure 3A), indicating that DHA had little effect on cell type markers. However, the GPMV assay revealed a clear stabilization of raft phases in both epithelial and mesenchymal states (Figure 3B). The raft stabilizing effect of DHA fully antagonized the reduction in PM phase separation induced by EMT. That is, when EMT was induced by over-expression of Snail in combination with DHA supplementation, the raft destabilizing effect of EMT was entirely abrogated. These results were confirmed in cells that had undergone EMT induced by TGF-β (Supplementary Figure 4).

Since DHA reversed the effects of EMT on plasma membrane organization and induced an epithelial-like biophysical membrane state, we tested whether this reversion would also enforce epithelial functional phenotypes, specifically lower rates of migration and stem cell characteristics. To test the effect of DHA-induced raft stabilization on cell migration, we used a scratch-wound assay. As expected, we observed that migration efficiency was significantly increased after an EMT; however, this increased migration was fully reversed by DHA treatment (Figure 3C). As another phenotypic characteristic associated with an EMT, we evaluated stem cell properties using a mammosphere formation assay [39]. Consistent with prior reports, we observed that EMT-transformed cells have a much higher potential for both primary and secondary mammosphere formation. However, like the increase in motility, this potential was also completely inhibited by DHA feeding, indicating a decreased ability both to form, and also to renew, mammosphere populations (Figure 3D).

Previous studies have found that tumor initiating cells in breast cancer exhibit a characteristic profile of the across cell surface markers CD44 and CD24. Differentiated luminal cells exhibit a CD44^−^CD24^+^ signature, while breast stem cells manifest as CD44^+^CD24^−/low^ [40]. CD24^−/low^ expression by itself has been associated with non-epithelial or basal stem cells [41]. In addition to expression changes, in an EMT, CD44 is alternatively spliced from a *variant* isoform (CD44v) in epithelial cells to a *standard* one (CD44s) in mesenchymal ones (Figure 3E) [42, 43]. Splicing to CD44s enables interaction with hyaluronic acid, activating TGF-β signaling among other pathways [44, 45]. This isoform switching is a critical attribute of the tumor initiating cell subpopulation [46].

We measured the profile of stem cell markers and confirmed that HMLE cells exhibit a differentiated signature, while ectopic expression of Snail leads to a CD44^+^CD24^−/low^ stem cell signature (Figure 3F), consistent with previous reports [5]. Treatment with DHA resulted in a CD44^+^CD24^+^ hybrid state, showing a loss of the CD24^−/low^ basal stem cell state, but no change in CD44 expression as detected by a pan-CD44 antibody that recognizes epitope 1 [40, 47]. To more specifically probe for changes in CD44 isoforms, we used RT-qPCR primers to probe for the CD44v (epithelial) and CD44s (mesenchymal) transcripts [43]. This revealed that snail expression led to a shift from the CD44v isoform to CD44s, consistent with an EMT, that was reversed with DHA treatment (Figure 3G). Although DHA resulted in no discernible change in CD44 protein expression, its splicing was altered.

Taken together, these results indicate that changes in the membrane organization are sufficient to alter the phenotypic consequences of an EMT, but do not reverse the expression of markers indicative of cell state.

### Raft stability coincides with stem cell phenotypes but not EMT state after slug knockdown

**Figure 4:**
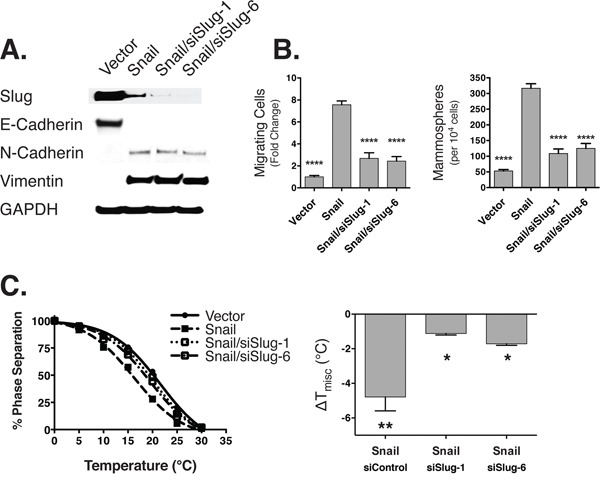
Slug knockdown leads to loss of stem cell properties concomitant with stabilization of raft domains **A.** This immunoblot shows the expression of slug (SNAI2), epithelial (e-cadherin), and mesenchymal (n-cadherin, vimentin) markers in HMLE cells with and without ectopic expression of Snail, and siRNA knockdown of slug. **B.** Under the same conditions, we measured (left panel) the fold change in cell migration (y-axis) using a transwell migration assay, as well as the (right) mammosphere forming capability of the cells. Statistical tests of significance are performed against the Snail sample as the baseline. **C.** (Left) These curves show the phase separation of the GPMVs across temperature. The differences relative to the vector control is summarized in the right panel.

We observed that rafts are destabilized in an EMT, and that restabilizing rafts with DHA interferes with stem cell properties but not mesenchymal markers. Therefore, we hypothesized that genetically ablating the drivers of stem cell properties specifically would, in turn, restabilize rafts regardless of cell state. To accomplish this, we induced EMT in HMLE cells with ectopic expression of snail. Then, we inhibited stem cell properties by knocking down slug, a transcription factor that is sufficient but perhaps not necessary for maintenance of a mesenchymal state, but is a master regulator of mammary stem cell activities [48–50]. In our snail-induced EMT models, we found that slug knockdown resulted in no detectable changes in the expression of e-cadherin, n-cadherin, or vimentin (Figure 4A). However, there was a marked decrease in cell migration and mammosphere formation (Figure 4B) concomitant with a stabilization of raft phase separation (Figure 4C). The fact that slug knockdown restabilized rafts, and reduced stem cell properties, in cells that maintained mesenchymal markers is consistent with our previous observation that PM organization is altered in response to changes in stem cell characteristics independently of cell state.

### DHA disrupts mesenchymal signaling pathways

Having observed that DHA supplementation inhibits phenotypes associated with an EMT—namely cell motility and stem cell characteristics—we next asked whether the impact of DHA was limited to these processes, or whether DHA-mediated membrane remodeling has a broader effect on the comprehensive transcriptional program of the cell. Using DNA microarrays, we measured the transcriptional profiles of HMLE cells under 6 conditions (Vehicle Control, Snail-induced EMT, TGF-β-induced EMT; each with and without 20 μM DHA treatment) and used GSEA to identify pathways that were induced in an EMT by comparing the control samples to the ones where EMT was induced [51, 52]. This analysis yielded 1030 pathways that were enriched in the mesenchymal conditions at a false discovery rate (FDR) < 25%, and 43 with reported FDR=0 (Supplementary Table 1). The most significant ones include many pathways clearly associated with mesenchymal cancer cells, including EMT signatures (ANASTASSIOU_CANCER_MESENCHYMAL_TRANSITION_SIGNATURE, CHARAFE_BREAST_CANCER_LUMINAL_VS_MESENCHYMAL_DN), CDH1 repression (ONDER_CDH1_TARGETS_2_UP), and stem cell properties (BOQUEST_STEM_CELL_UP, LIM_MAMMARY_STEM_CELL_UP).

**Figure 5:**
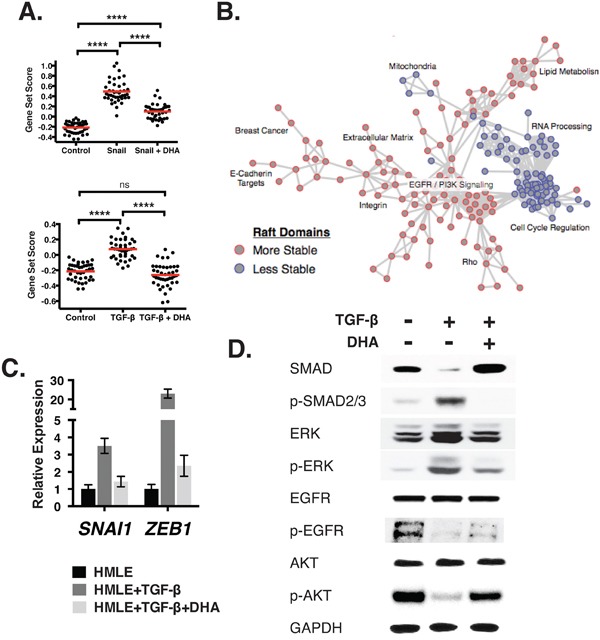
Alterations in membrane organization lead to transcriptional reprogramming of the cell **A.** Gene sets for pathways associated with an EMT state are scored (y-axis) across samples with and without EMT inducers (Snail, top; TGF-β, bottom), and with and without DHA treatment (x-axis). Scores are relative, and higher scores indicate stronger evidence for activation of the pathway. **B.** This network shows the relationship between the pathways (shown as nodes) associated with stable (red nodes) or destabilized (blue) raft domains. The labels summarize the pathways that comprise dense hubs within the network. **C.** Relative gene expression (y-axis) of inducers of EMT *SNAI1* and *ZEB1* after treatment with 2.5 nM TGF-β and 20 μM DHA. **D.** Immunoblots show the expression of cell signaling proteins after treatment with TGF-β and DHA.

To test whether DHA can perturb the 43 highly significant EMT-associated pathways, we scored the expression profiles of EMT-induced cells fed with DHA (Figure 5A). While EMT-associated pathways were induced by snail, this effect was significantly attenuated upon treatment with DHA. In the case of EMT induced by TGF-β, DHA-treated scores were statistically indistinguishable from those of epithelial cells. Thus, DHA treatment resulted in a widespread inhibition of mesenchymal-related gene expression profiles.

Because DHA showed broad changes in the transcriptional profiles, we performed an unbiased analysis to identify the pathways that are associated with destabilization of membrane domains. To do this, we again applied GSEA, but now comparing the samples with more stable raft domain separation in GPMVs (Control, DHA, Snail+DHA, and TGF-β+DHA) against the ones with destabilized raft domains (Snail and TGF-β). Using Enrichment Map, we generated a network of pathways significantly affected by PM domain organization (Figure 5B) [53]. This analysis confirmed that alterations in raft stability resulted in changes in motility-related molecules such as Rho, extracellular matrix, and integrins. In addition, it revealed widespread metabolic programming, including changes in lipid metabolism and RNA processing. Finally, alterations in proliferative events were notable, including changes in EGFR signaling and cell cycle regulation.

To verify the *in silico* pathway predictions, we measured the transcript levels and confirmed an increase in the expression of *SNAI1* and *ZEB1*, direct repressors of *CDH1* transcription [54, 55] (Figure 5C). To measure changes in the activation of signaling pathways, we generated immunoblots and confirmed that TGF-β treatment led to an increase in phosphorylation of SMAD2/3, a major effector of the pathway (Figure 5D). This was accompanied by increased ERK phosphorylation, as predicted by the *Cell Cycle* annotations seen in the microarray analysis. Also consistent with this analysis is the loss in *EGFR / PI3K Signaling*, as measured by EGFR and Akt phosphorylation. Finally, each of these changes were reversed or attenuated by the addition of DHA. Altogether, DHA feeding led to changes across a wide range of cellular processes related to EMT, demonstrating the importance of PM organization in cancer progression.

### DHA abrogates lung colonization *in vivo*

**Figure 6:**
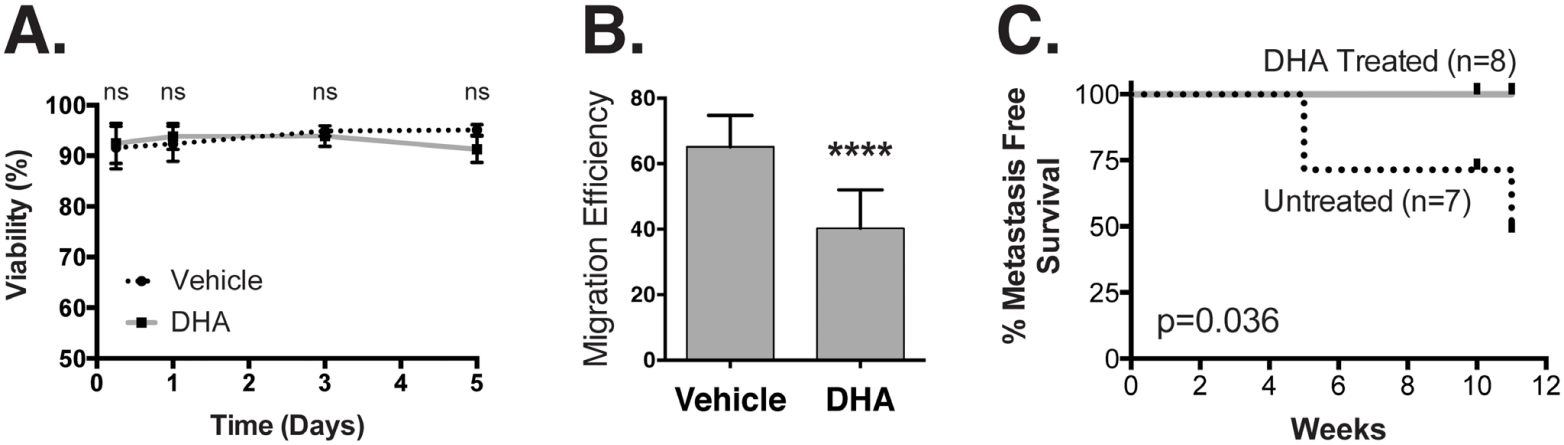
DHA treatment inhibits lung metastasis **A.** We measured the viability of MDA-MB-231/pMir-Luc cells using a Trypan blue exclusion test after treatment with 20 μM DHA for up to five days. **B.** DHA treatment decreases migration efficiency in a scratch-wound assay. **C.** 1×10^6^ MDA-MB-231/pMir-Luc cells pre-treated with vehicle or 20 μM DHA for five days were injected into the lateral tail vein of immunocompromised NOD/SCID mice. This Kaplan-Meier survival curve shows the time to detection of lung metastasis (x-axis) via bioluminescence imaging.

The observation that perturbations in PM organization disrupt cell motility and stem cell pathways suggests that this organization may be useful for targeting the metastatic process. To evaluate this possibility, we used an experimental metastasis assay to determine whether PM organization can impact colonization, the most clinically viable target in the metastatic cascade [56]. As a model, we chose a claudin-low breast cancer cell line MDA-MB-231/pMIR-Luc that has the capacity to metastasize to the lung [57]. These cells express luciferase, which we leveraged to monitor the development of lung nodules. *In vitro*, cells treated with 20 μM DHA for 5 days showed no change in cell viability (Figure 6A), although there was a significant decrease in migration efficiency, confirming that they respond to DHA in a similar fashion to the HMLE line (Figure 6B). Next, we injected 1×10^6^ cells intravenously into the lateral tail vein of immunocompromised NOD/SCID mice and monitored for metastases for up to 11 weeks via bioluminescence imaging. We noted the time at which metastases first appeared, and saw that the treated cells had no observable metastatic potential (Figure 6C). Treatment of the cells with DHA abrogated the ability of the cells to colonize the lung, demonstrating an impact on metastatic potential *in vivo*.

## DISCUSSION

Our study investigated the relationship between the organization of the PM and EMT and found that induction of EMT reduces phase separation in isolated plasma membrane vesicles, indicative of less stable raft domains. The experimental capability to measure raft phase stability has only recently been developed, and used to measure exogenous mediators of PM biophysical properties [21]. Bile acids, lipid binding proteins, and liquid anesthetics have all been shown to regulate the temperature at which microscopic phase separation can be observed in isolated PMs [21, 23, 24]. The structural and compositional principles underlying this regulation of PM organization are currently not well understood.

In contrast to previous studies [21, 24], our observations focused on cell-autonomous alterations of membrane organization. The robust biophysical changes we observed are almost certainly driven by the modification of the composition of the PM. Recent advancements in comprehensive lipidomics have revealed a startling complexity and flexibility of membrane lipid composition in both mammalian and fungal cells [13, 58]. Specifically, in an *in vitro* EMT model, dramatic lipidomic alterations were observed, with downregulation of canonical raft lipids induced by transition to the mesenchymal phenotype [13]. Such changes are consistent with our observations of increased PM fluidity induced by EMT [15], though it must be emphasized that the actual relationships between composition and physical properties in complex, protein-rich membranes like GPMVs are unknown and unlikely to be easily predictable.

The observation that transcriptional changes precede the biophysical membrane alterations implies that reduced raft stability is not necessary for the initiation of EMT. However, the altered PM organization appears to be an integral characteristic of the metastatic phenotype, as (a) EMT induced by four distinct means consistently reduced raft phase separation; (b) conversely, MET increased raft phase separation; (c) stabilizing raft phase separation by genetic or pharmacologic means reversed two important phenotypic changes induced by EMT (motility and mammosphere formation); (d) stabilizing rafts inhibited EMT-associated signaling networks; and (e) stabilizing rafts impaired the ability of cells to colonize the lung. Thus, we conclude that alterations of PM biophysical phenotypes are required to maintain metastatic potential and can decouple the mesenchymal state from the stem cell phenotype.

The relationship between PM organization and EMT is consistent with the hypothesized central role of PM domains in regulation of cell signaling [59]. Although this role has primarily been investigated in immune cells [60], rafts have also been widely implicated in cancer signaling [61]. Moreover, a number of key signal transduction molecules have been shown to be regulated by the organization of the PM, most notable among these being the Ras GTPases which integrate signals from a variety of pathways to induce cell growth, proliferation, and survival [62]. Although the mechanistic details of raft involvement in most signaling pathways remain unresolved, it is hypothesized that rafts behave as PM micro-compartments that tune the output of signaling reactions by selectively recruiting specific components and excluding others [63].

Although we have yet to dissect the signaling programs modulated by PM organization in our model, the demonstration that specific PM biophysical properties are important for EMT suggests that this important oncogenic process may be targeted by unconventional treatments aimed at inhibiting the PM changes associated with EMT. Indeed, alkylphospholipid drugs (i.e. miltefosine and edelfosine) that have been described as *dis-rafters* for their apparent ability to affect raft organization have potent antineoplastic activity [27]. In this context, our observation that the essential dietary ω-3 fatty acid DHA stabilizes raft domains and thereby antagonizes the EMT-induced PM phenotype is particularly intriguing. It suggests that the PM phenotype is subject to dietary inputs, and therefore long-term perturbations in organismal lipid phenotypes (e.g. hypercholesterolemia, DHA supplementation) may have previously unrecognized effects on cell signaling and pathogenesis.

## MATERIALS AND METHODS

### Reagents

DHA was purchased from Sigma as a pure oil in a purged, sealed ampoule. PUFAs have a tendency to oxidize when exposed to air or aqueous solution as free fatty acids, so DHA was complexed with BSA immediately upon opening. To this end, 1 mM BSA solution (in ddH2O) was added to DHA to produce a 2:1 FA:BSA solution. The resulting solution was stirred for 2 h at room temp to produce BSA-DHA complexes which were then sterile filtered, aliquoted, and stored under N_2_ at −80°C. Recombinant human TGF-β (R&D Systems #240-B) was dissolved in sterile 4 mM HCl with 1 mg/mL BSA at 20 μg/mL.

### Ectopic gene expression

Bicistronic, retroviral vector pWZL-hygro (addgene plasmid #18750) was used as the backbone for expression of Snail and Twist. Snail and Twist were cut from pBabe-puro backbone previously containing the genes using BamHI (New England Biolabs #R0136) and EcoRI (New England Biolabs #R0101) restriction enzymes. Slug.fl was cut from pPGS-hSLUG.fl.flag (Addgene plasmid #25696) with EcoRI. pWZL was cut with the corresponding restriction enzymes for each insert. The inserts were ligated using T4 DNA ligase (New England Biolabs #M0202) overnight at 16°C. Inserts were verified using restriction digest followed by size verification on 1.4% agarose gel and outside sequencing (Lone Star Labs).

Deacylated PEI transfection reagent [64] (a generous gift from Dr. Guangwei Du, University of Texas Health Sciences Center at Houston) was used to transfect 4 μg plasmid DNA into PLAT-A packaging cells grown to roughly 30% confluence. HMLE and T47D cells were infected for 3 hours with viral media after 48 hours of packaging and again after 72 hours. Cells positive for incorporation of either empty pWZL, or pWZL containing Snail, Twist, or Slug were selected with media containing 5 μg/ml Hygromycin B (Thermo Fisher Scientific #10687).

### Lentiviral infection and FOXC2 knockdown

To suppress FOXC2 expression, we used the shRNA-expressing pLKO lentivirus system. We purchased from Sigma-Aldrich pLKO.1-puro vectors containing shRNA targeting either no known mammalian genes (#SHC002), or *FOXC2* (shFOXC2-3 clone ID TRCN0000015385, shFOXC2-5 clone ID TRCN0000015386). The production of lentiviruses and infection of target cells were followed as previously described [15]. The stable suppression of FOXC2 was achieved by selection of cells in 2 μg/ml of puromycin.

### siRNA transfection

Cells were transfected with siRNA against human slug (Sigma-Aldrich #SASI_Hs01_00159363, #SASI_Hs01_00159364) and siRNA universal negative control (Sigma-Aldrich #SIC002) using Lipofectamine RNAiMAX transfecting agent (Thermo Fisher Scientific #13778) according to manufacturer's instructions. We performed reverse transfection to knockdown expression of slug, using 50 nM siRNA and 1.5% Lipofectamine RNAiMAX. Cells were incubated with RNAi duplexes for 48 h before collection for analysis.

### Cell culture and EMT induction

HMLE cells were grown in a 1:1 ratio of DMEM:F12 (Thermo Fisher Scientific #11320-033) and MEBM + Bullet Kit (Lonza # CC-3150). T47D cells were gown in RPMI (Thermo Fisher Scientific #SH3002701) with 10% FBS (VWR #83007-198) and 1% Pen Strep (Thermo Fisher Scientific #10378-016).

We induced an EMT in HMLE or T47D cells by incubation with 2.5 ng/mL TGF-β, or ectopic expression of EMT-inducing transcription factors Snail, Slug, or Twist. Cells stably expressing these transcription factors, as well as ER-Snail, were a gift from the Sendurai Mani lab at The University of Texas MD Anderson Cancer Center. 4-Hydroxytamoxifen (Sigma-Aldrich #H7904) was added at a final concentration of 20 nM to ER inducible Snail HMLE cells.

We performed scratch-wound, transwell migration, and mammosphere formation assays as previously described [15].

### GPMV isolation and imaging

Briefly, cells were grown to 60-80% confluence, then washed in GPMV buffer (10 mM HEPES, 150 mM NaCl, 2 mM CaCl_2_, pH 7.4, in dH_2_O). Cell membranes were then stained with 5 μg/mL of FAST-DiO (Thermo Fisher Scientific #D-3898), a lipidic dye that strongly partitions to L_d_ phases due to double bonds in its fatty anchors. Then, GPMVs were extracted by adding a solution of GPMV buffer + 25 mM PFA + 2 mM DTT and incubated at 37°C for 2 hours (HMLE) or 1 hour (T47D). The GPMV-containing solution was collected and mounted on BSA-coated coverslips for imaging.

The mounts were attached to a Peltier/temperature controlled microscope stage and imaged with green florescent laser under 40x objective. At each condition, 50-100 vesicles were counted as either phase separated or phase combined.

Increasing the temperature of the vesicles causes miscibility of the two phases. We raised the temperature in 3°C increments, at every increment counting each vesicle as either phase separated or phase combined (Figure 1A, Supplementary Figure 2). We continued these measurements until nearly all GPMVs were no longer microscopically phase separated. We then evaluated the change in temperature at which 50% of the GPMVs were phase separated by fitting to a sigmoid curve.

### Real time quantitative PCR

mRNA samples were isolated with Qiagen's RNeasy Mini Kit (#74106) according to manufacturer's protocol with DNAse digestion. Reverse transcriptase PCR was performed on these samples using the Verso cDNA Synthesis Kit (#AB-1453) from Thermo Fisher Scientific according to manufacturer's protocol. To quantify mRNA expression, SYBR Fast MasterMix (2x) Universal Dye (#KK4602) from Kapa Biosystems was used in an Eppendorf Realplex^2^ Mastercycler. Each primer set for each sample was run in triplicate with 1 ng of cDNA per well.

**Table:** 

Gene	Forward Primer	Reverse Primer
E-cadherin	GTCACTGACACCAACGATAATCCT	TTTCAGTGTGGTGATTACGACGTTA
Claudin 1	CCTATGACCCCAGTCAATGC	TCCCAGAAGGCAGAGAGAAG
N-cadherin	ACAGTGGCCACCTACAAAGG	CCGAGATGGGGTTGATAATG
Vimentin	CCAAACTTTTCCTCCCTGAACC	GTGATGCTGAGAAGTTTCGTTGA
CD44V	GCTCATACCAGCCATCCAAT	GAGGTCCTGTCCTGTCCAAA
CD44S	CCCTGCTACCAATAGGAATGAT	TTCAGATCCATGAGTGGTATGG
Snail	CCTCCCTGTCAGATGAGGAC	CCAGGCTGAGGTATTCCTTG
ZEB1	CCGAGCCTCCAACTTTACCT	TAGAGGCTCTCGCTCTACGG

### Immunoblotting

Immunoblots were conducted as previously described [15]. We used the following antibodies:

**Table:** 

Protein	Catalog #	Dilution Ratio
Slug	Cell Signaling #9585	1:1000
E-Cadherin	Cell Signaling #3195	1:6000
N-Cadherin	Santa-Cruz #sc-53488	1:1000
Vimentin	Cell Signaling #5741	1:3000
GAPDH	Cell Signaling #5174	1:6000
Smad	Santa-Cruz #sc-7153	1:1000
Phospho-Smad2/3	Cell Signaling #8828	1:1000
ERK1	Santa-Cruz #sc-94	1:1000
Phospho-ERK	Santa-Cruz #sc-7383	1:1000
EGF Receptor	Cell Signaling #4267	1:1000
Phospho-EGF Receptor	Cell Signaling #2231	1:1000
Akt1/2/3	Santa-Cruz #sc-8312	1:2000
Phospho-Akt	Cell Signaling #9271	1:1000

### Flow cytometry

CD24 and CD44 expression were analyzed in cells derived from monolayer cultures harvested by brief incubation 0.05% trypsin/0.025% EDTA. Detached cells were washed and resuspended (10^6^ cells/100 μl) in HBSS buffer (Thermo Fisher Scientific #14175-095) containing 10 mM HEPES (Thermo Fisher Scientific #15630080) and 2% FBS. Then the cells were stained with the fluorochrome-conjugated primary antibody against human CD44 (APC; BD Biosciences #559942) and CD24 (FITC; BD Biosciences #555427) or their respective isotype controls at concentrations recommended by the manufacturer, and incubated at 4°C in the dark for 30 min. After that, the labeled cells were washed in HBSS buffer containing 25 mM HEPES and analyzed on a BD LSRFortessa Cell Analyzer System. The FlowJo software package was used to analyze data.

### Microarray analysis

mRNA samples were isolated with Qiagen's RNeasy Mini Kit (#74106) according to the manufacturer's protocol with DNAse digestion. 100 ng of each sample was hybridized on the Illumina HumanHT-12 v4 Expression BeadChip (#BD-103-0204) and scanned at the UTHSC Quantitative Genomics & Microarray Core Lab. We pre-preprocessed the gene expression data using the IlluminaExpressionFileCreator module in GenePattern [65], followed by quantile normalization [66].

We performed pathway analysis using the GSEA module available at GenePattern [51, 52]. We analyzed against the C2 curated gene sets in the MSigDB database version 5.0 and selected the most significant ones with FDR=0 [67]. Then, we scored the activation of those gene sets against each of the samples. To make sure that genes represented by multiple probes on the array are not overweighted in the score, we selected as a representative probe for each gene the one with highest variance. Then, we normalized each gene so that the mean expression across all samples was 0 and variance was 1. Next, we used this processed data to derive a single score for each gene set. To account for the fact that gene sets in MSigDB are separated into UP and DOWN pairs (i.e. the genes whose expression increase in a pathway, and those that decrease), we negated the expression of the genes in the DOWN gene set so that in both cases, high scores would signify activation of the gene set. To derive the score, we averaged the expression of each gene in the pair of gene sets.

We performed network analysis with EnrichmentMap [53]. The network consists of the gene sets with significance *p* < 0.005 and FDR < 0.05. Edges connect pairs of nodes with Overlap Coefficient > 0.5.

### Noninvasive bioluminescence imaging of experimental lung metastases *in vivo*

Breast cancer cells stably expressing luciferase (MDA-MB-231/pMir-Luc) were a gift from the Sendurai Mani lab at The University of Texas MD Anderson Cancer Center. Cells were grown in DMEM (Thermo Fisher Scientific #11995-065) supplemented with 10% FBS and were either treated with 20 μM DHA or vehicle for 5 days. Then, cells were trypsinized, centrifuged, resuspended in 1x PBS, pH 7.4 and counted. 1×10^6^ cells in 200 μl of PBS were injected into the lateral tail vein of 10-week old female immunocompromised NOD/SCID mice (NOD.CB17-Prkdcscid/J; The Jackson Laboratory, Bar Harbor, ME). Eight mice were injected with DHA-treated cells and seven mice were injected with untreated cells. Mice were monitored for lung metastases from 5 to 11 weeks after injection of cells using digital camera attached to computer with Living Image 4.4 software (IVIS Lumina XR; PerkinElmer, Waltham, MA). IVIS captures luminescence in the living animal when luciferase in MDA-MB-231/pMir-Luc cells catalyzes the oxidation of luciferin, which emits photons. Mice were anesthetized with isoflurane/oxygen, and then luciferin (VivoGloTM Luciferin, *In vivo* Grade; Promega, Fitchburg, WI) was injected into the intraperitoneal cavity at 120 mg/kg (24 mg/ml). Immediately after injection, images of the ventral side were collected for 1 min. Bioluminesce in the lung region represents lung metastases. To determine the percentage of lung metastasis free survival, we monitored the time when metastases first appeared and performed Kaplan-Meier analysis using GraphPad Prism.

All animal experiments were performed in compliance with the protocols approved by the Animal Welfare Committee of the Center for Laboratory Animal Medicine and Care at The University of Texas Health Science Center at Houston.

## SUPPLEMENTARY FIGURES AND TABLE




