# Supplementary material for: Motility and stem cell properties induced by the epithelial-mesenchymal transition require destabilization of lipid rafts
Source: Oncotarget. 2016 Jun 9;7(32):51553–68. doi: 10.18632/oncotarget.9928 (PMC5239496; doi:10.18632/oncotarget.9928)
Supplement: Supplementary file 1 [file oncotarget-07-51553-s001.pdf]

Motility and stem cell properties induced by the epithelial-mesenchymal transition require destabilization of lipid rafts

SUPPLEMENTARY FIGURES AND TABLE

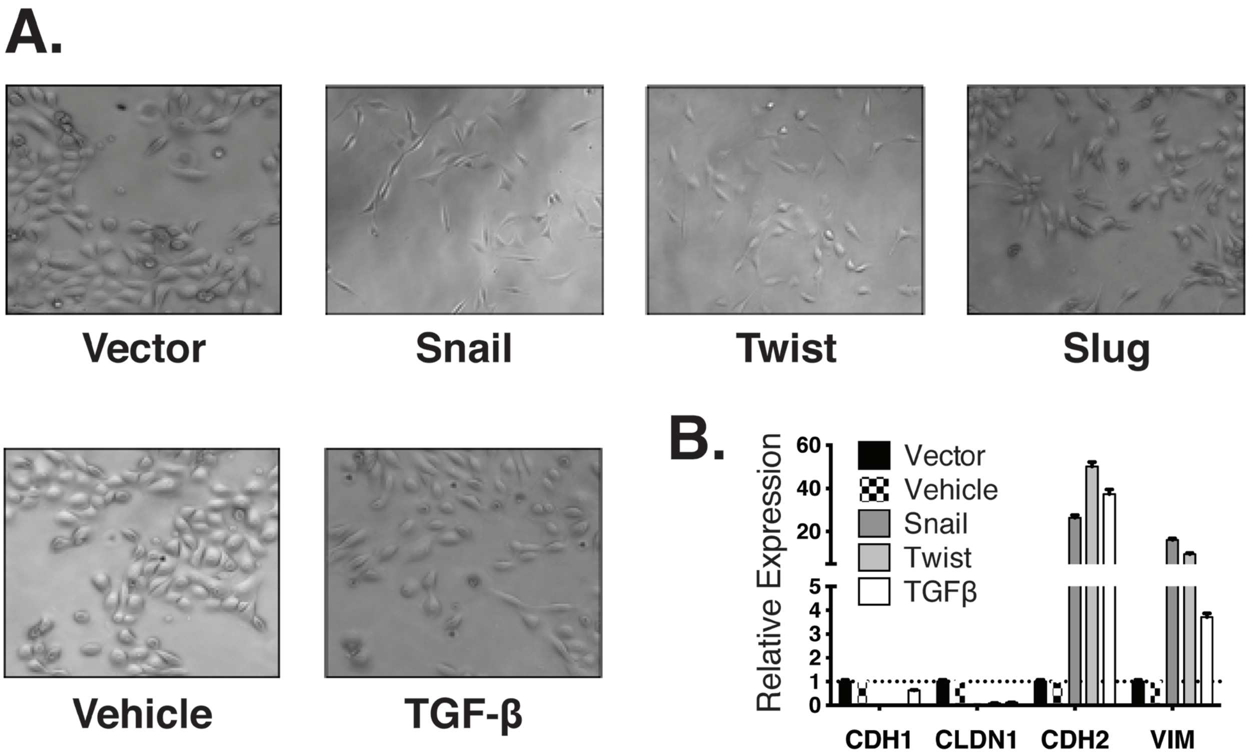

Supplementary Figure S1: EMT can be induced by a range of mechanisms. A. Brightfield images show the morphology of HMLE cells after ectopic expression of EMT inducers Snail, Twist, or Slug; or one week treatment with TGF-β. **B.** The gene expression values (y-axis) for epithelial (CDH1, CLDN1) and mesenchymal (CDH2, VIM) are measured by RT-QPCR in HMLE cells before and after induction of an EMT. All gene expression changes are significant with $p < 0.05$ when compared to the appropriate Vector or Vehicle control.

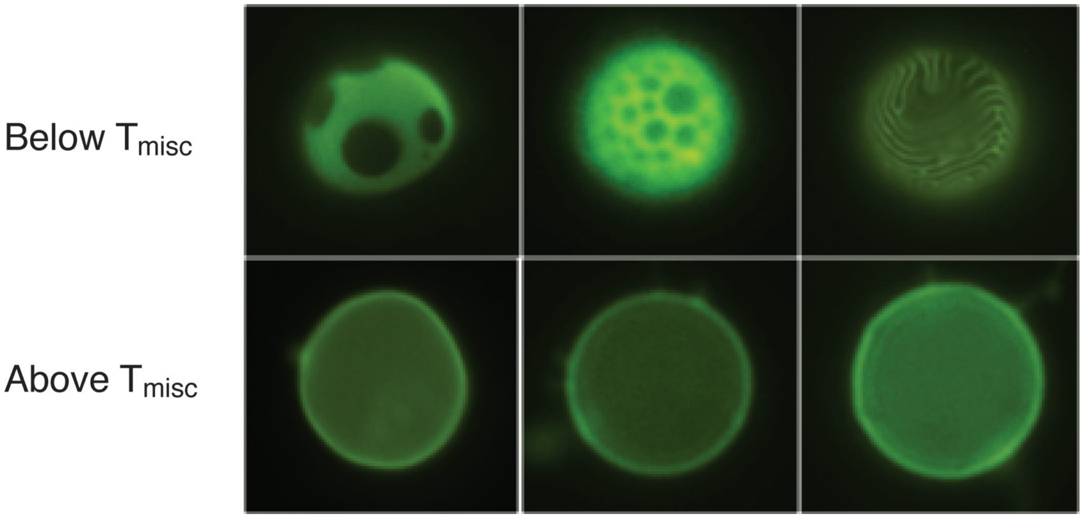

Supplementary Figure S2: Distinct patterns of phase separation in GPMVs. GPMVs show clear phase separation above T_{misc}, and no separation below it.

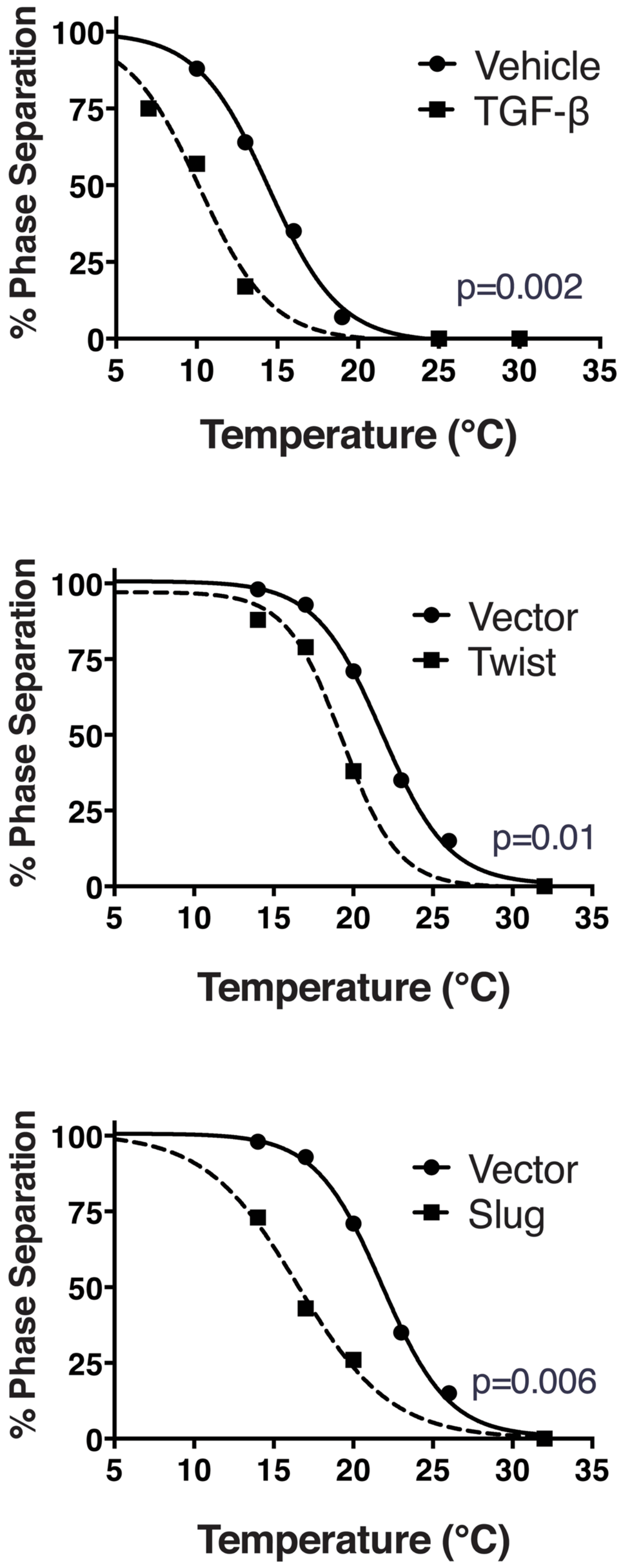

Supplementary Figure S3: EMT inducers destabilize phase separation. These plots show the relationship between the stability of phase separation (y-axis) across temperature (x-axis) for EMT inducers TGF-β, Twist, or Slug.

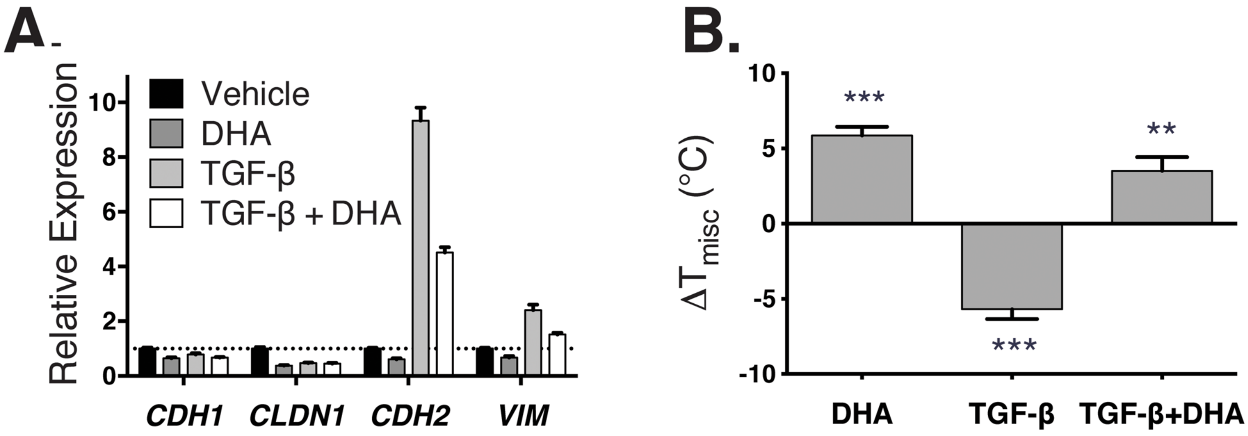

Supplementary Figure S4: DHA treatment maintains a mesenchymal state while reversing raft stability. A. The gene expression values (y-axis) for epithelial (CDH1, CLDN1) and mesenchymal (CDH2, VIM) are measured by RT-QPCR in HMLE cells treated with a vehicle control (*Vehicle*), 20 µM DHA (*DHA*), 2.5 ng/mL TGF-β (*TGF-β*), or both TGF-β and DHA (*TGF-β+ DHA*). All expression values differ from the Vehicle control with $p < 0.05$. **B.** The raft stability (y-axis) for the treatments is shown after normalization to the Vehicle condition.

Supplementary Table S1: GSEA analysis identifies pathways enriched in an EMT with FDR=0

See Supplementary File 1
